# Dynamics of glutamatergic signaling in the mushroom body of young adult *Drosophila*

**DOI:** 10.1186/1749-8104-5-10

**Published:** 2010-04-06

**Authors:** Irina Sinakevitch, Yves Grau, Nicholas J Strausfeld, Serge Birman

**Affiliations:** 1 Laboratoire de Neurobiologie, CNRS UMR 7637, ESPCI ParisTech, 10 rue Vauquelin, 75231 Paris cedex 5, France; 2 Institut de Biologie du Développement de Marseille-Luminy, CNRS UMR 6216, Université de la Méditerranée, Campus de Luminy case 907, 13288 Marseille cedex 9, France; 3 Institut de Génomique Fonctionnelle, CNRS UMR 5203, INSERM U661, Université de Montpellier I et II, 141 rue de la Cardonille, 34094 Montpellier cedex 5, France; 4 Center for Insect Science and Department of Neuroscience, University of Arizona, Tucson, Arizona 85721, USA; 5 Current address: Arizona State University, School of Life Sciences, Tempe, AZ 85287-4501, USA

## Abstract

**Background:**

The mushroom bodies (MBs) are paired brain centers located in the insect protocerebrum involved in olfactory learning and memory and other associative functions. Processes from the Kenyon cells (KCs), their intrinsic neurons, form the bulk of the MB's calyx, pedunculus and lobes. In young adult *Drosophila*, the last-born KCs extend their processes in the α/β lobes as a thin core (α/β cores) that is embedded in the surrounding matrix of other mature KC processes. A high level of L-glutamate (Glu) immunoreactivity is present in the α/β cores (α/βc) of recently eclosed adult flies. In a *Drosophila *model of fragile X syndrome, the main cause of inherited mental retardation, treatment with metabotropic Glu receptor (mGluR) antagonists can rescue memory deficits and MB structural defects.

**Results:**

To address the role of Glu signaling in the development and maturation of the MB, we have compared the time course of Glu immunoreactivity with the expression of various glutamatergic markers at various times, that is, 1 hour, 1 day and 10 days after adult eclosion. We observed that last-born α/βc KCs in young adult as well as developing KCs in late larva and at various pupal stages transiently express high level of Glu immunoreactivity in *Drosophila*. One day after eclosion, the Glu level was already markedly reduced in the α/βc neurons. Glial cell processes expressing glutamine synthetase and the Glu transporter dEAAT1 were found to surround the Glu-expressing KCs in very young adults, subsequently enwrapping the α/β lobes to become distributed equally over the entire MB neuropil. The vesicular Glu transporter DVGluT was detected by immunostaining in processes that project within the MB lobes and pedunculus, but this transporter is apparently never expressed by the KCs themselves. The NMDA receptor subunit dNR1 is widely expressed in the MB neuropil just after eclosion, but was not detected in the α/βc neurons. In contrast, we provide evidence that DmGluRA, the only *Drosophila *mGluR, is specifically expressed in Glu-accumulating cells of the MB α/βc immediately and for a short time after eclosion.

**Conclusions:**

The distribution and dynamics of glutamatergic markers indicate that newborn KCs transiently accumulate Glu at a high level in late pupal and young eclosed *Drosophila*, and may locally release this amino acid by a mechanism that would not involve DVGluT. At this stage, Glu can bind to intrinsic mGluRs abundant in the α/βc KCs, and to NMDA receptors in the rest of the MB neuropil, before being captured and metabolized in surrounding glial cells. This suggests that Glu acts as an autocrine or paracrine agent that contributes to the structural and functional maturation of the MB during the first hours of *Drosophila *adult life.

## Background

The neurotransmitter L-glutamate (Glu) plays essential roles in various brain functions in mammals, such as motor control, synaptic plasticity, learning and memory, cognition, and brain maturation during development [1-5]. Disruption of Glu signaling is central to epilepsy [6,7] and major neurological and psychiatric disorders, including Alzheimer's and Parkinson's diseases, schizophrenia, mood disorders, depression, anxiety, and stress- and trauma-related disorders [3,8-10]. Glu acts by binding to specific ion channel-coupled ionotropic (iGluRs) or G protein-coupled metabotropic (mGluRs) membrane receptors. Glu receptors are implicated in processes of learning and memory through long-term potentiation, a form of synaptic strengthening that follows brief, high frequency stimulation [11-14] and long-term depression, a long lasting reduction in synaptic transmission [12,15,16]. Glu release from nerve endings or astrocytes [17,18] requires previous uptake and concentration in synaptic vesicles by vesicular Glu transporters [19,20]. However, Glu can also be released by non-vesicular mechanisms [21] and exerts a paracrine action on neuronal migration [22,23].

In *Drosophila *and other arthropods, Glu is well characterized as the excitatory neurotransmitter of the neuromuscular junction [24-28]. However, this amino acid has important signaling functions in the *Drosophila *brain as well [29-33]. The *Drosophila *genome was predicted to encode 30 iGluR subtypes, including 3 AMPA- and 15 kainate-like, 2 NMDA-like, 4 δ-like and 6 divergent receptors [34]. For now, the best characterized of these are the postsynaptic iGluRs expressed at the neuromuscular junction [25]. *Drosophila *NMDA-like receptors are expressed in the central nervous system [35] and have been implicated in learning and memory [36] and locomotor control [37]. The *Drosophila *genome encodes a single functional mGluR, DmGluRA, an ortholog of vertebrate group II mGluRs [38]. This mGluR is presynaptic and expressed at the periphery of the active zones at the glutamatergic neuromuscular junctions, where it modulates both synapse excitability and fine structure [24]. DmGluRA is also expressed in the brain, in particular in lateral clock neurons, where it regulates circadian locomotor behavior [39].

The mushroom bodies (MBs) are paired centers located in the protocerebrum of *Drosophila *and other dicondylic insects that play essential roles in olfactory learning and memory [40] and other brain functions, such as the control of locomotor activity [41], courtship behavior [42], courtship conditioning [43], visual context generalization [44], and sleep [45]. The intrinsic structure of the MB is provided by the Kenyon cells (KCs), which have their cell bodies in the brain cortex and their dendrites in the MB calyx, where they receive input from the antennal lobe projection neurons. Axon-like processes of KCs project anteriorly and ventrally in the peduncle to form the vertical and medial lobes, which are subdivided into discrete parallel entities, the vertical α, α' and the medial β, β' and γ lobes. In addition to the KCs, there are other MB intrinsic neurons and several classes of MB extrinsic neurons that connect the MB to other areas of the brain neuropil [33,46-48]. Emerging evidence suggests that different subtypes of MB KCs may be involved in distinct mechanisms of memory formation due to their connections to different MB extrinsic neurons [49-53].

Developmental studies have shown that the KCs are produced in each hemisphere of the brain by the division of four neuroblasts born early during the embryonic stage. The division of these neuroblasts sequentially produces the three morphologically and spatially distinct subtypes of KCs: γ, α'/β' and α/β [54,55]. The γ neurons are generated up to the mid-third instar larval stage; they form the larval dorsal and medial lobe [55,56]. The next KC subtype to be generated is the α'/β' neuron, which continues to be produced until puparium formation. Lastly, the α/β neurons are generated from the time of puparium formation until adult eclosion. In the α/β lobes, the KCs are organized in concentric layers. The youngest axon-like processes situated in the inner layer of the lobes are successively displaced outwards as they differentiate and newer α/β processes are added to the structure from the most recently born KCs [47]. This volume of the α/β lobes into which grow the last-born axons contains densely packed and extremely thin fibers that are rich in actin filaments. This subset of processes has been named the α/β core (α/βc) [33,47].

An increased response to mGluR activation may play a prominent role in the fragile X syndrome (FXS), the most common form of inherited mental retardation and the predominant cause of autism [57]. Mutations in *dFmr1*, the *Drosophila *homologue of the gene implicated in FXS, lead both to learning deficits and altered development of the MB, of which the most common feature is a failure of β lobes to stop at the brain midline [58]. These behavioral and developmental phenotypes can be successfully rescued in *Drosophila *by treatment with mGluR antagonists [59], implicating Glu in the pathology, as is the case in mammalian models [60]. Recent studies showed that dFmr1 interacts with DmGluRA in the regulation of synaptic architecture and excitability at glutamatergic synapses [61,62]. However, until now the precise role of Glu and mGluRs in FXS and MB development has remained obscure.

Here we present evidence that Glu and its receptor DmGluRA are directly involved in construction of the MB neural circuits. Previous studies suggested that the *Drosophila *last-born α/βc KCs are immunoreactive to anti-Glu antibodies [32,33]. In the present study, we show that these neurons express a high level of Glu-like immunoreactivity in newly eclosed adult flies. Interestingly, newborn KCs in late larval and pupal stages also appear to express as a rule a high level of Glu. To understand further the role and fate of Glu during KC maturation, we analyzed the dynamics of Glu, DmGluRA and other Glu signaling-associated proteins in the MB of young adult *Drosophila *from the time of their eclosion until 10 days post-eclosion. Our results indicate that a transient Glu release likely regulates functional maturation of newborn KCs by a paracrine action during *Drosophila *post-embryonic development and the first hours after adult eclosion.

## Results

### High Glu levels in last-born Kenyon cells

**Figure 1:**
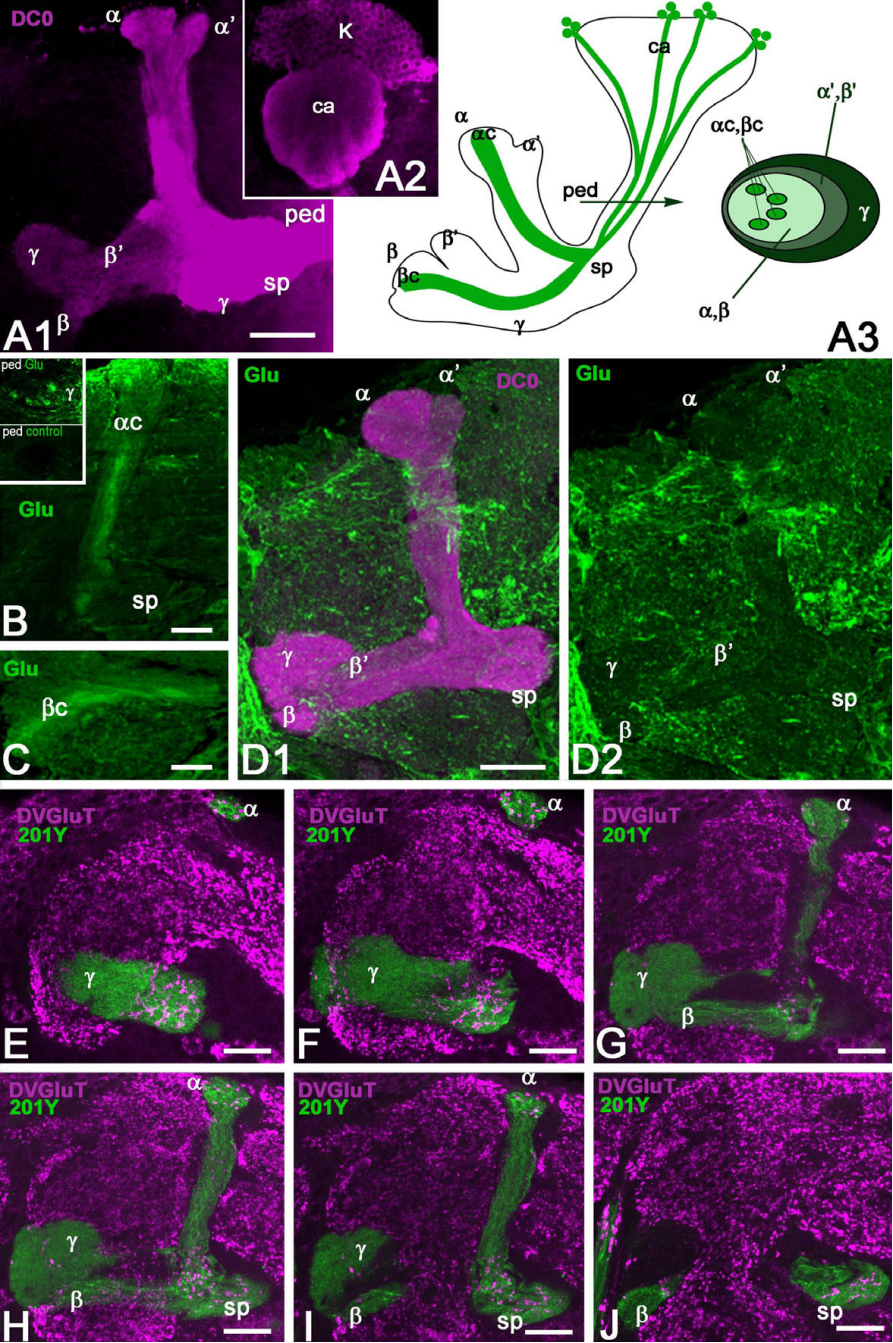
**Glutamate and DVGluT immunoreactivities in the *Drosophila *mushroom bodies after adult eclosion**. **(A1, A2) **DC0 marks the whole MB neuropil that consists of the calyx (ca) and pedunculus (ped), and the vertical (α, α') and horizontal (β, β' and γ) lobes. The spur (sp) area of the pedunculus contains axons of the γ lobe. **(A3) **Schemes of the adult MB just before eclosion. The last born KCs extend axons in the cores of the α and β lobes (αc, βc). Arrow indicates the position of a transverse section of the pedunculus on the right showing the layered and concentric organization of the KC axons. **(B, C) **Frontal sections of the MB lobes labeled with anti-Glu antibodies. One hour after adult eclosion, Glu can be detected at a high level in the lobes but only in the αc and βc axons. Upper insert in (B) shows a transverse section of the pedunculus with strong Glu staining in the α/βc KCs. The lower insert is the control for Glu immunostaining after preabsorption of the diluted antibodies with 10^-4 ^M conjugated Glu. **(D1, D2) **In 10-day-old flies, Glu immunoreactivity (green) is absent from the DC0-stained KCs (magenta). Glu is distributed at this stage in scattered patterns in the α and γ lobes and in the spur (sp), and likely originates from MB extrinsic neurons. **(E-J) **DVGluT immunoreactivity in the MB within 1 hour after after eclosion. Confocal optical sections from the frontal to the posterior parts of the protocerebrum from 201Y-GAL4; UAS-mCD8::GFP flies. Note that the intrinsic α/βc KCs are not positive for DVGluT. Stained processes from extrinsic neurons are found in the α and γ lobes and in the spur (sp) region. Scale bars: 20 μm.

Diverse subtypes of KCs form the *Drosophila *MB neuropil and their axons extend in the pedunculus and in the medial and vertical lobes [33,48]. The whole *Drosophila *MB structure can be revealed with anti-DC0 (PKA-C1) antibodies that label all parts of the KCs with different intensity (Figure 1A1, A2): the cell bodies (K), the dendrites in the calyx (ca), and the axons in the pedunculus (ped) and lobes. In the γ lobe, KCs extend only one axonal branch, forming a medial lobe and the spur (sp), whereas the axons of other KCs divide at the level of the spur to give rise to vertical and medial axonal branches, thus forming the α'/β' and α/β divisions of the lobes. The KC axons are organized in concentric strata in the pedunculus and α/β lobes. This is schematically depicted in Figure 1A3, the first-born cells, that is, the γ neurons, being the most external, and the last-born cells, the α/βc neurons, being embedded within the surrounding processes of α/β neurons. Like the other KCs, these last-born α/βc neurons originate from four neuroblasts, which provide four identical axonal bundles in the pedunculus and throughout the MBs (Figure 1A3).

The α/βc KCs present a high level of Glu-like immunoreactivity in their newborn axons immediately after adult eclosion (Figure 1B, C). Figure 1B (insert) shows that Glu-like immunoreactivity in the pedunculus is restricted to four thin axonal bundles of α/βc KCs embedded within the α/β axons. In contrast, no Glu-like immunoreactivity can be detected in the older neurons in the core of the α/β lobes in 10-day-old adult flies (Figure 1D1, D2). At this age, we observed Glu-like immunoreactivity scattered in the α and γ lobes and in the spur. This pattern is likely to represent the distribution of Glu-like immunoreactivity in the processes of MB extrinsic neurons rather than in KCs.

### Last-born Kenyon cells do not express DVGluT

The accumulation of Glu in the young α/βc KCs suggests that this amino acid could be transiently used as a neurotransmitter by these cells. The vesicular Glu transporter DVGluT is involved in Glu synaptic vesicle storage, prior to neurotransmitter release, and can be used as a marker of glutamatergic neurons in *Drosophila *[29,30]. Therefore, we performed anti-DVGluT immunostaining immediately after adult eclosion, at a time when the Glu level is high in the MB core neurons. The DVGluT antibodies strongly labeled the protocerebrum and antennal lobe neuropils. In contrast, in the MB neuropil, neither the core neurons nor any intrinsic KCs were found to be immunopositive for DVGluT (Figure 1E-J). The DVGluT immunoreactivity observed in the MBs, particularly in the spur of the pedunculus and in the γ and α lobes (Figure 1E-J), most likely corresponds to synapses from extrinsic glutamatergic neurons. This suggests that Glu in the young α/βc KCs either is not stored in a vesicular pool or is stored in vesicles by another transporter not yet identified.

### MB driver expression in last-born KCs

**Figure 2:**
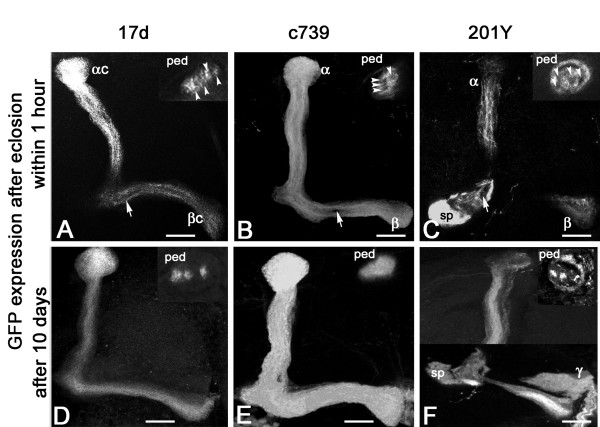
**Commonly used mushroom body drivers do not target the last born Kenyon cells just after adult eclosion**. **(A) **In 17d-GAL4; UAS-mCD8::GFP adults just after eclosion, GFP is not present in the inner axons of the α/βc neurons as seen in the lobe (arrow) or in the pedunculus (arrowheads in the insert). **(B) **Ten days later, all the α/βc KCs express GFP as shown in the lobes and pedunculus (insert). **(C, D) **Similar observations with the c739-GAL4 driver line that does not express GFP in the α/βc neurons right after eclosion (C), but target all α/β KCs 10 days later (D). sp, spur. **(E, F) **Similar observations with the 201Y-GAL4 driver line that targets both the α/β and γ neurons. This line does not target the last born α/βc KCs just after eclosion as well. Scale bars: 20 μm.

The previous results suggest that the last-born MB core neurons mature within just a few days after eclosion from an early Glu-expressing state to a differentiated cell in which Glu no longer accumulates. We asked whether other MB markers could be used to differentiate between these two maturation states. The enhancer trap lines 17d-, c739-, and 201Y-GAL4 mimic the expression of MB markers and are commonly used as MB-specific drivers. We crossed each of these lines to UAS-mCD8::GFP flies to characterize their expression pattern in young adults. When flies were collected immediately after eclosion, we found every time that the Glu-expressing α/βc neurons consist of two populations: inner α/βc neurons, which corresponds to the younger cells that do not express green fluorescent protein (GFP); and outer α/βc neurons, which do express GFP (Figure 2A-C and inserts). In contrast, in 10-day-old flies, we observed that these three drivers express mCD8-GFP in all the α/βc KCs: 17d-GAL4 in the α/βc neurons only (Figure 2D), c739-GAL4 in the α/β and α/βc cells (Figure 2E), and 201Y-GAL4 in the α/βc and γ lobe neurons (Figure 2F). In each of these lines, GFP immunostaining arranges in a specific way revealed by the pedunculus sections shown in the Figure 2 inserts: 17d-GAL4 expresses GFP in four inner bundles corresponding to the α/βc cells, c739-GAL4 in the whole area of the pedunculus that contains the α/β axons, and 201Y-GAL4 in the four α/βc bundles and a peripheral surrounding area that contains the γ lobe axons. Thus, at least three different MB markers are not expressed in the immature last-born α/βc KCs at a time when these cells show high levels of Glu immunoreactivity, while these markers are strongly expressed in these same cells in 10-day-old flies.

### Rapid disappearance of Glu from last-born Kenyon cells

**Figure 3:**
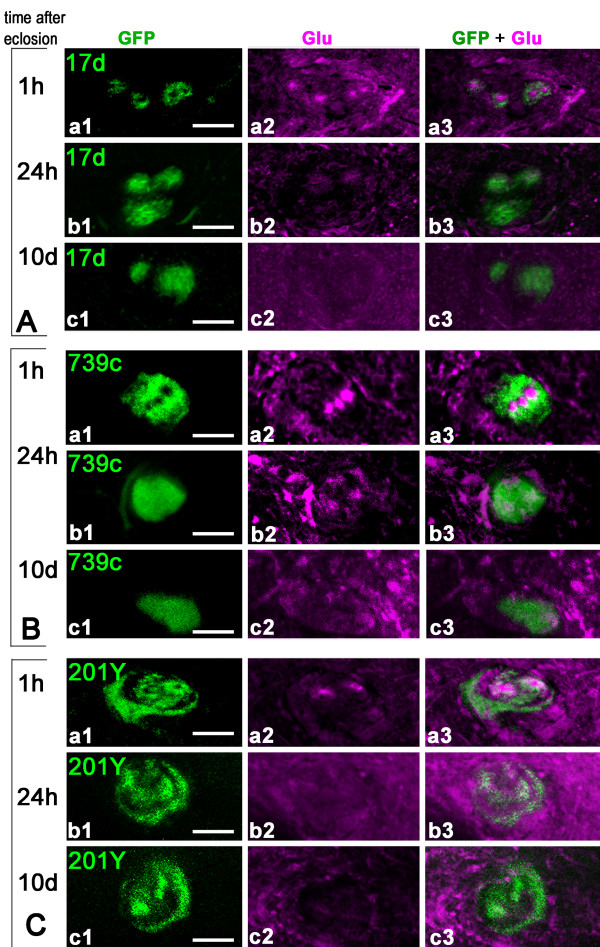
**Rapid disappearance of Glu immunoreactivity from the mushroom body α/β cores following adult eclosion**. **(A-C) **Transverse sections of the MB pedunculus from 17d-GAL4 (A), c739-GAL4 (B) and 201Y-GAL4 (C) adult flies expressing mCD8::GFP and labeled for Glu at different ages. Similar results were obtained with the three driver lines. **(A-C, a1-a3) **Within 1 hour after eclosion, the core neurons express Glu but not GFP. Only thin processes in the outer layers of the pedunculus are positive for both. **(A-C, b1-b3) **Twenty-four hours after eclosion, the intensity of Glu labeling already dramatically decreased while the expression of GFP started to appear in the core neurons. **(A-C, c1-c3) **Ten days after eclosion, there is no detectable Glu in the core neurons and GFP is strongly expressed in these cells. Scale bars: 10 μm.

We then precisely compared the temporal patterns of Glu and GFP expression in the α/βc KCs of MB driver lines expressing mCD8-GFP by looking at the pedunculus in frontally cut agarose sections (Figure 3). In all three GAL4 lines (17d-, c739-, and 201Y-GAL4) early after eclosion, the α/βc neurons could be divided into three subtypes: the most inner α/βc neurons that express Glu but not GFP; more peripheral α/βc neurons that co-express glutamate and GFP; neurons in the α/βc outer area that express only GFP. Within 1 hour after eclosion, only the outer parts of the α/βc region were simultaneously positive for both GFP and Glu in each driver line (Figure 3A-C, panels a1-a3). Because the border of the core regions co-localizes with GFP, this suggests that the maturing α/βc neurons begin to express GFP at the time when Glu is still present. Twenty-four hours after eclosion, GFP starts to be expressed in most α/βc neurons. This is when Glu-like immunoreactivity was already becoming dramatically reduced (Figure 3A-C, panels b1-b3). Ten days after eclosion, there is no detectable Glu immunoreactivity in the α/βc KCs, which all express GFP at a high intensity (Figure 3A-C, panels c1-c3). Therefore, maturation of the last-born KCs appears to be a fast process in *Drosophila *that is concluded in a few hours only after adult eclosion.

**Figure 4:**
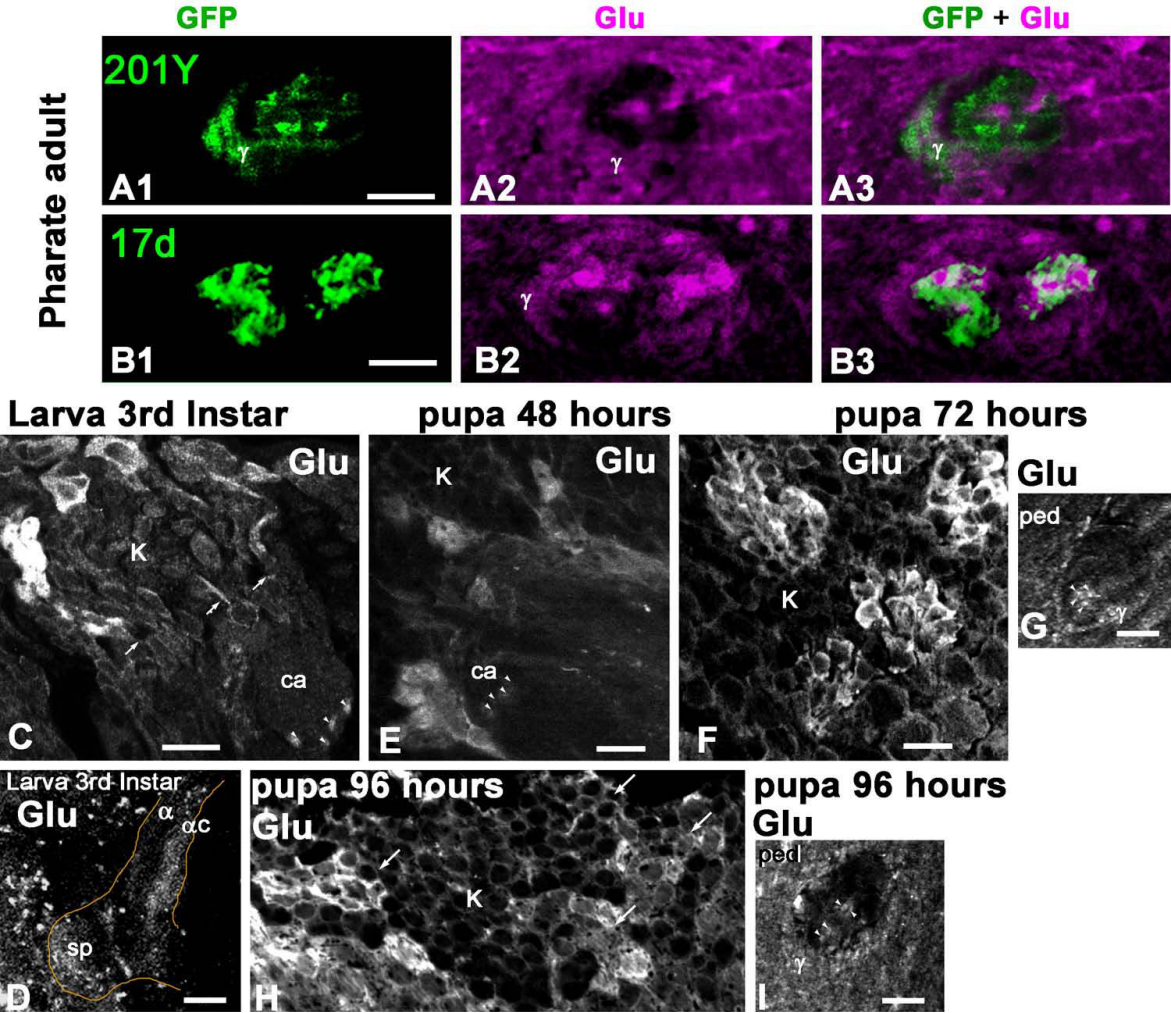
**Newborn Kenyon cells express high levels of Glu in the *Drosophila *mushroom body in late larval stage and during pupal development**. **(A, B) **Transverse section of the MB pedunculus from 201Y-GAL4 (A) and 17d-GAL4 (B) pharate adults collected a few hours before eclosion and expressing mCD8::GFP. **(A1-A3) **GFP and Glu expression in 201Y-GAL4 are located both in the α/βc and γ lobes. Most of the GFP-positive α/βc neurons do not co-localize with Glu and only few axons express both Glu and GFP at the border of Glu-containing a/βc neurons. Agarose section of the pedunculus close to the lobes. **(B1-B3) **In 17d-GAL4, most Glu-containing α/βc neurons do not express GFP and only the border axons are both GFP- and Glu-positive. Agarose section of the pedunculus close to the calyx. In (A, B), Glu immunoreactivity in the γ lobe most likely originates from extrinsic neurons. **(C-I) **Glu immunoreactivity monitored at late larval and pupal stages in the MB of wild-type *Drosophila*. (C, D). Transverse sections through the calyx and lobes of wandering third instar larva. The four bundles of Glu-positive processes originate from four clusters of KCs. Only one cluster is shown (on the left side in (C)) and arrows show bundles originating from the other Glu-positive clusters. The four Glu-positive bundles extend in the core area of the pedunculus (arrowheads), indicating that they correspond to newborn KCs. As shown in (D), these Glu-positive fibers also project into the α/βc region of the larval MB (βc is not visible on this agarose section). The contour indicates the shape of the larval MB vertical lobe (α) and spur (sp) region. Note that extrinsic glutamatergic cells are also present at this stage in the spur area. (E) In pupa 48 hours after puparium formation (APF) at 22°C (stages P5 to P6 of Bainbridge and Bownes [100]), clusters of Glu-positive KCs are still present but their bundles at the base of the pedunculus (arrowheads) are less intensely labeled compared to larval and other pupal stages. (F, G) Pupa 72 hours APF (stage P8). At this developmental stage, four groups of newborn KCs are brightly Glu-positive and project axons in the pedunculus (arrowheads in (G)). Other Glu-immunoreactive processes in the γ lobe area of the pedunculus likely correspond to fibers from extrinsic cells as only cell bodies of newborn KCs express high Glu-immunoreactivity. (H, I) In pupa 96 hours APF at 22°C (stages P11 to P12), four clusters of Glu-immunopositive newborn KCs (arrows in (H)) project their axons to the pedunculus (arrowheads in (I)). K, Kenyon cell bodies; ca, calyx, ped, pedunculus; sp, spur. Scale bars: 10 μm.

We observed that Glu is already present in the α/βc axons of pharate adults, removed from the pupal cases a few hours before eclosion (Figure 4A, B). At this stage, Glu immunoreactivity is also detectable in the γ region of the MB pedunculus. Representative pictures are shown in Figure 4A1-A3, section close to the lobes, and Figure 4B1-B3, section close to the calyx. The MB drivers 201Y-GAL4 and 17d-GAL4 do not express GFP in the Glu-expressing α/βc neurons of pupal flies, except for a few neurons at the border of the core. In 201Y-GAL4, GFP is also present in the γ area in part of the pedunculus. The level of Glu immunoreactivity in the γ lobe varied significantly amongst flies sampled before eclosion, probably depending on the exact developmental progress of each pupal animal. This signal probably originates from extrinsic glutametergic neurons because we found that only cell bodies of newborn KCs express high Glu-immunoreactivity (see Figure 4C, I and text below).

### Newborn Kenyon cells express Glu during larval and pupal stages

In order to explore if Glu is similarly present in developing KCs during earlier phases of MB development, we performed Glu immunostaining on brain agarose sections in late third instar larva and at various stages of pupal development. At these stages, we generally found four clusters of Glu-immunoreactive KC bodies that send four Glu-positive bundles in the pedunculus and lobes (Figure 4C, I). Strikingly, these Glu-positive bundles are always located in the core areas of the pedunculus (arrowheads in Figure 4C, E, G, I), indicating that they correspond to newborn cells. A Glu signal was also detected in the γ lobe area in late pupae (Figure 4G) that most likely originates from extrinsic neurons as the only Glu-positive KC bodies are those of newborn cells. Therefore, although more work is needed to draw a precise picture of Glu expression in MB during metamorphosis, it seems to be a rule that developing KCs transiently express high levels of Glu during the formation and/or maturation of their neural circuits in the *Drosophila *MB.

### Mushroom body-associated glial cells express glutamine synthetase

Because we suspected that the Glu accumulated in the last-born immature KCs could be released and play a role in KC maturation, we looked for expression of further glutamatergic markers in the vicinity of the α/βc in newly eclosed flies. In mammalian nervous systems, subtypes of glial cells are equipped with specific proteins to ensure Glu recapture, metabolism and recycling. These include high affnity Glu transporters [63,64] and the enzyme glutamine synthetase (GS), which converts the captured Glu into glutamine [65-67]. Such glial cells can extend cytoplasmic processes close to Glu neurotransmitter release sites. Similarly, in the *Drosophila *central nervous system, the cell surface Glu transporter dEAAT1 is expressed by peripheral glia of the cell body cortex and addressed to glial processes that invade the neuropil proper [31], and the fly glutamine synthetase orthologue GS2 is expressed in glial cell subsets [68-70] (T Rival and S Birman, unpublished).

**Figure 5:**
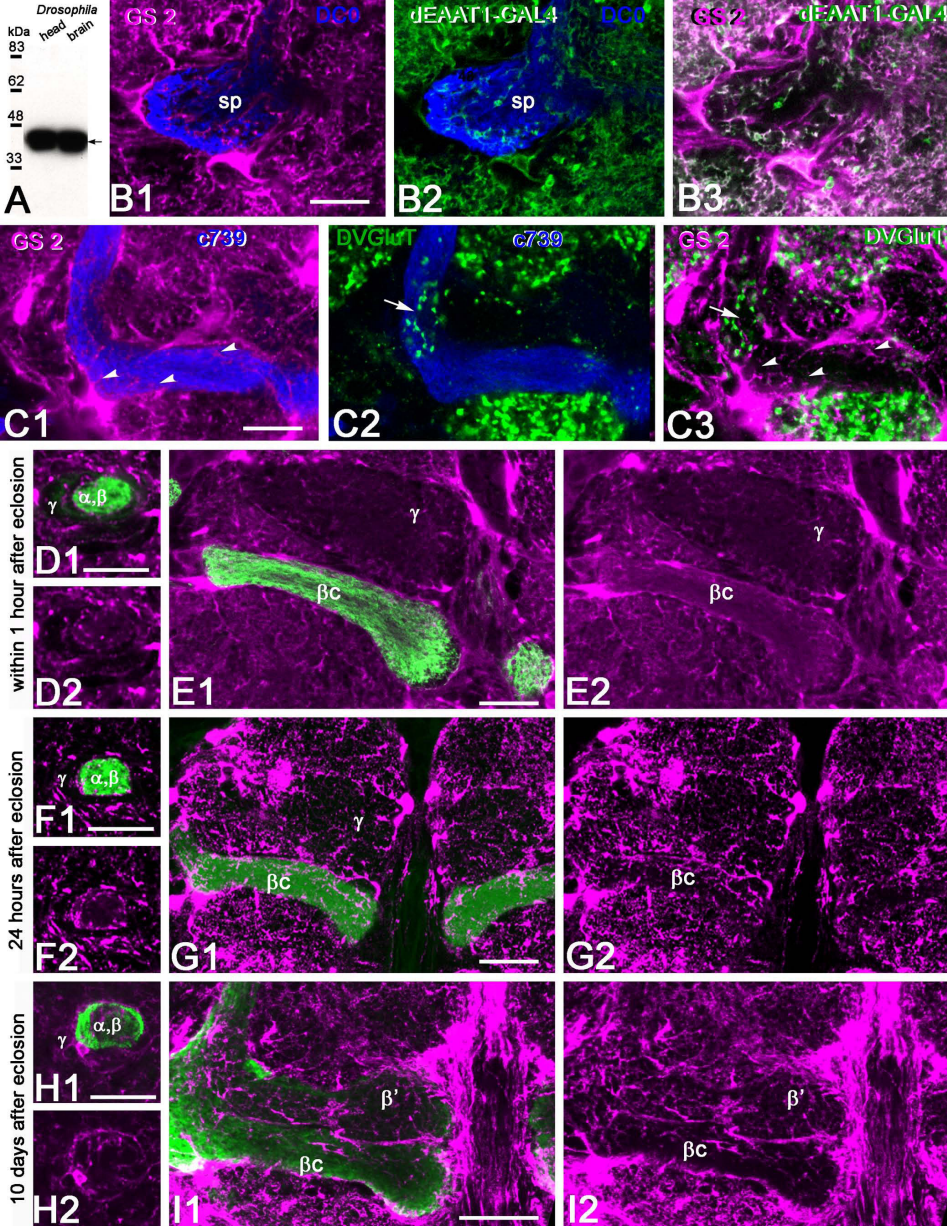
**Dynamics of glutamine synthetase expression in mushroom body glial cells after adult eclosion**. **(A) **A mouse monoclonal antibody raised against sheep glutamine synthetase (GS) recognizes a single band in a western blot of *Drosophila *head and brain. The apparent size of the protein (approximately 42 kDa) closely corresponds to the predicted size of *Drosophila *GS2 (41 kDa). **(B) **Localization of GS immunoreactivity in glial cells that express the Glu transporter dEAAT1 in 10-day-old adult fly MBs. Triple staining of brains from dEAAT1-GAL4; UAS-dEAAT1::GFP *Drosophila *with anti-GS (magenta), anti-GFP (green) and anti-DC0 (blue, for MB staining). **(B1-B3) **GS co-localizes with dEAAT1-positive glial cell bodies and processes in the spur (sp) region of the MB neuropil (merged magenta and green produces a white color). **(C) **Localization of GS in the MB with respect to glutamatergic synapses. Triple staining of wild-type adult brains with anti-DVGluT (green), anti-GS (magenta), and anti-GFP (blue) in agarose sections. **(C1) **GS is distributed all over the lobes (arrowheads) except the core neurons. **(C2) **DVGluT-positive glutamatergic synapses are present on the α lobe (arrow). **(C3) **GS immunoreactivity lies in close vicinity to glutamatergic synapses (arrow). In the β lobe, GS is present (arrowheads) but DVGluT is absent. **(D-I) **Sections of *Drosophila *brains stained with anti-GS (magenta) at different adult ages showing the MB pedunculus (D, F, H) and the medial lobe region (E, G, I). MB α/β KCs are positive for GFP (green) in c739-GAL4; UAS-mCD8::GFP flies (D-G) or DC0 (green) in wild-type flies (H-I). In the pedunculus, GS is found in glial cells that surrounds the α/β axons either within 1 hour after adult eclosion (D), 24 hours later (F) or at 10 days old (adult) (H). (E) Within 1 hour after eclosion, GS-immunoreactive processes enwrap the MB medial β and γ lobes. GS is also detected in many profiles inside the β and γ lobes, except in the regions corresponding to the core neurons that express neither GFP nor GS. (G) Twenty-four hours after eclosion, GS-positive glial processes distribute evenly in the whole β lobe area, including the β core, which at this time expresses GFP. (I) Ten days after eclosion, GS is expressed at a high level in glial cells that surround the β lobes. Scale bars: 20 μm.

A monoclonal antibody raised against sheep brain GS recognizes only one band on western blots of *Drosophila *brain proteins, which migrates close to the predicted size of GS2 (Figure 5A). *In situ *immunolabeling revealed that the fly GS is present in dEAAT1-expressing glial cells that surround the whole MB (Figure 5B). These glial cells also extend elaborate velate process inside the MB neuropil (Figure 5B, C). We observed in our preparations that GS-positive glial processes primarily surround the α/β neurons and enwrap their axons in the pedunculus and lobes, separating them from the other (γ, α'/β') divisions of the lobes. These glial processes originate from cells that lie on the surface over the MBs as well as between the lobes, thereby enclosing and isolating the MB from the surrounding protocerebral neuropils. Co-immunolabeling against GS and DVGluT showed that some GS-positive glial processes lie close to glutamatergic synapses, particularly in the α lobes (Figure 5C). However, a large network of GS-positive glial processes can be observed throughout the entire MB neuropil, including volumes such as the β lobe where DVGluT-positive processes are obviously absent (Figure 5C).

### Dynamics of glutamine synthetase expression during mushroom body maturation

We found that GS expression is highly dynamic in the MB region during the first days of *Drosophila *adult life. The network of GS-positive glial profiles in the α/β lobe area becomes progressively dense and elaborated with time. Immediately after eclosion, GS is expressed by glial cells that surround the whole MB and send processes into it. These glial processes branch into the α/β region of the pedunculus and lobes and envelop the α/β axons. Surprisingly, no glial branching occurs in the α/βc areas; these are merely surrounded by the GS-positive processes in newly eclosed flies (Figure 5D, E).

Twenty-four hours after eclosion, glial cell processes also begin to invade the core neuropil of the pedunculus and α/β lobes. Processes also distribute equally throughout the lobes, with the highest density of processes at the lobe's periphery. The GS-expressing glial cells form a thick layer that surrounds the α/β lobes and generate a fine mesh-like network everywhere inside these lobes, including the α/βc area (Figure 5F, G). In 10-day-old flies, glial cells express high levels of GS immunoreactivity and surround all the MB lobes, forming much more elaborate and extensive distributions that in newly eclosed flies (Figure 5H, I).

### NMDA receptor localization in the mushroom body region

**Figure 6:**
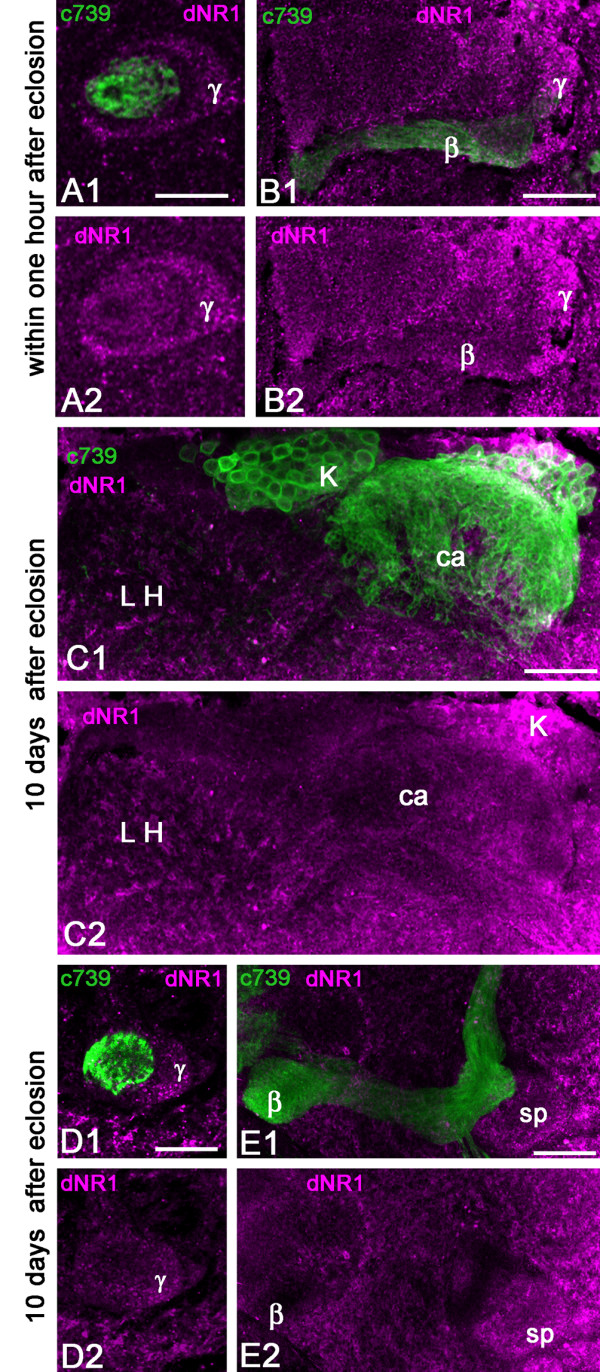
**Localization of the NMDA receptor subunit dNR1 in the mushroom body of young adult *Drosophila***. Double stainings with anti-dNR1 (magenta) and anti-GFP (green) in brain sections of c739-GAL4, UAS-mCD8::GFP flies. **(A, B) **In the MBs of recently eclosed flies, dNR1 is detected in processes of the pedunculus and lobes corresponding to the α/β and γ cells, whereas the core region is not labeled. **(C) **In the MBs of 10-day-old flies, some KC bodies are positive for dNR1 (K) but the calyx (ca) shows a low level of immunoreactivity. In contrast, a high level of dNR1 is present in the lateral horn (LH) area where the projection neurons from the antennal lobes end. **(D, E) **In 10-day-old flies, anti-NR1 staining is scattered within the α/β and γ areas of the pedunculus (D) and with high intensity in the spur (sp) region of the MB (E). Scale bars: 20 μm.

Next, we searched for the presence and distribution of Glu receptors potentially involved in learning and memory in the *Drosophila *MB. The NMDA receptors (NMDARs) are subtypes of iGluRs composed of an essential NR1 subunit and variable NR2 subunits. We applied antibodies against the *Drosophila *dNR1 protein on whole-mount or frontal agarose sections of the fly brain. The immunostaining on whole mounts revealed a brightly labeled cluster of neurosecretory neurons, in agreement with a previous report [36]. Additionally, we found dNR1 staining in the MB lobes that was only detectable on agarose sections. In newly eclosed flies, significant dNR1 immunoreactivity was observed in the MB pedunculus and lobes, with a higher level of staining in the γ axons, specifically on the surface neurons that make up the spur region of the MB neuropil. In contrast, the α/βc neurons were not stained with these antibodies (Figure 6A, B).

The dNR1 immunostaining appeared somewhat different in flies examined 10 days after eclosion (Figure 6C-E). The lateral horn of the protocerebrum exhibited a high level of dNR1 immunoreactivity. Some KCs express dNR1 but the MB calyx showed only sparse staining, suggesting that the NMDAR subunit is mainly addressed to cell bodies and axons of KCs invading any of the MB lobes except those formed by the α/βc neurons (Figure 6C-E). We observed that the staining in the MB lobes and pedunculus was dramatically reduced in 10-day-old flies compared to the staining of the same areas in flies collected within 1 hour after eclosion. The spur area of the pedunculus had the highest level of dNR1 immunoreactivity (Figure 6E). Interestingly, the spur region is also the area of the MB that receives the highest density of DVGluT-immunoreactive profiles (Figure 1E-J). The dNR1-positive processes might also be associated with extrinsic neurons of the MBs.

### DmGluRA-GAL4 transiently expresses in last-born Kenyon cells

**Figure 7:**
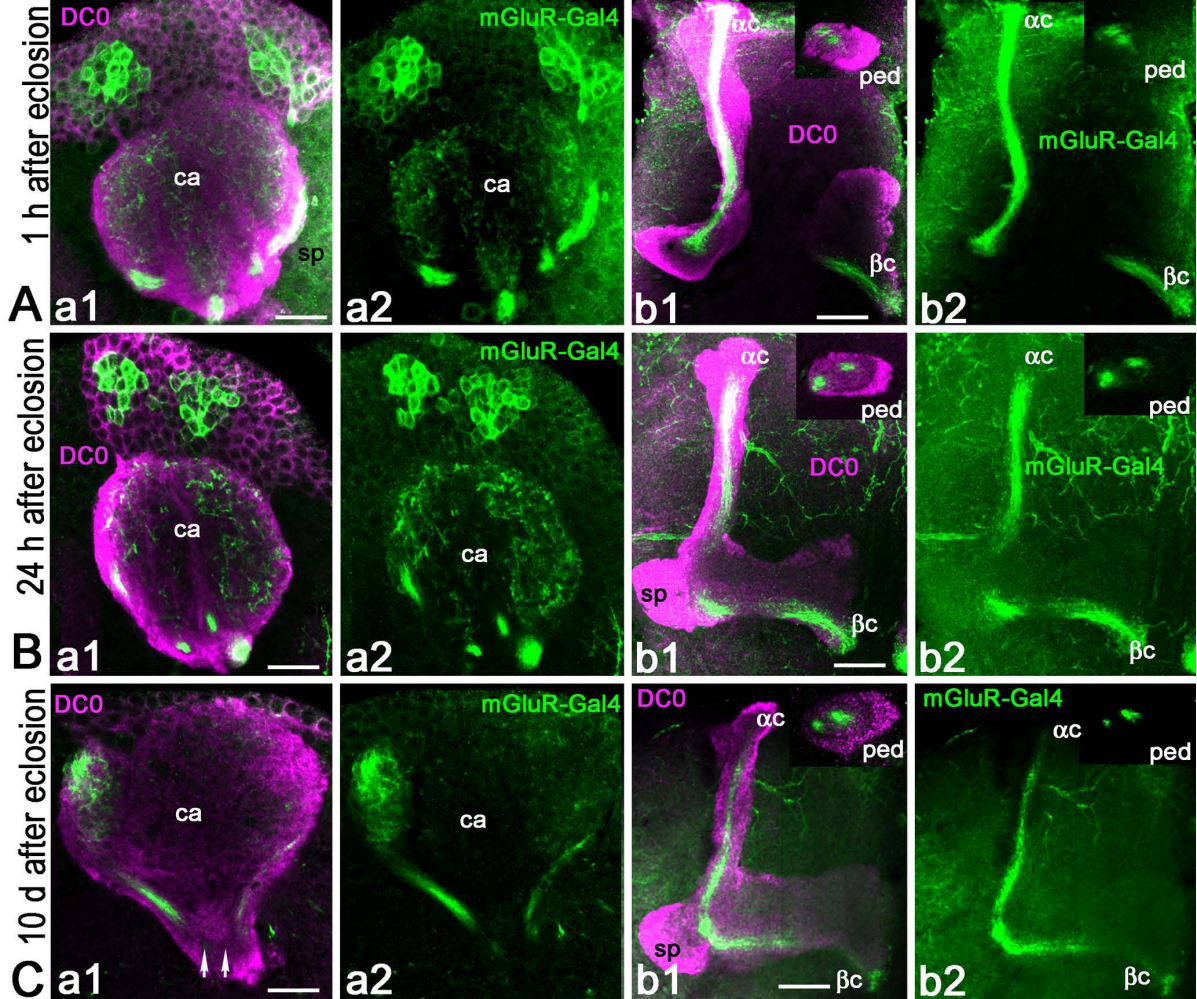
**DmGluRA-GAL4 expresses selectively in the mushroom body core neurons**. Double stainings for DC0 (magenta) and GFP (green) in brain sections of DmGluRA-GAL4, UAS-mCD8::GFP young adult flies. **(A) **Within 1 hour after eclosion, anti-GFP brightly stains the α/βc KCs, revealing their cell bodies and dendrites in the calyx (ca) (Aa) and their axons in the pedunculus (ped) (insert in (Ab)) and lobes (Ab). GFP is apparently also present in outer areas of the lobes. **(B) **Twenty-four hours after eclosion, the intensity of anti-GFP staining appears still high in the α/βc KCs. **(C) **Ten days after eclosion, GFP expression is less intense but still present in the α/βc neurons of the *Drosophila *MB. Not all axons from α/βc KCs but only two out of four clonal units are stained (arrows show the absence of the GFP in two groups of cells). So only part of the staining is maintained in the calyx (Ca) whereas intensity appears to still be high in the pedunculus and lobes (Cb). sp, spur. Scale bars: 20 μm.

DmGluRA is the only G-protein-coupled mGluR in *Drosophila*. This receptor has been shown to localize in the presynaptic site at the neuromuscular junction [24] and it is also expressed in the brain - for example, in clock neurons, where it regulates circadian locomotor behavior [39]. The GAL4 enhancer trap technique is a widely used method for analyzing tissue-specific gene expression patterns in *Drosophila*. To generate the insertion of a *GAL4*-containing *P *element in the regulatory region of the DmGluRA gene, localized on the fourth chromosome, we used a targeted transposition strategy as described by Sepp and Auld [71]. This technique induces the precise replacement of one *P *element for another. Starting from the line 39C42, in which an outmoded *P *element lacking any expression reporter is inserted 5.94 kb upstream of the translation initiation codon of DmGluRA [24], we generated a new strain in which this *P *element was replaced with a *P(Gal4) *enhancer trap element, here called *DmGluRA-GAL4*. We used this driver line to monitor the expression of the DmGluRA receptor in the MBs, with mCD8::GFP as a reporter gene. Strikingly, we found that the last-born KCs located in the inner part of the α/βc neurons express GFP in their cell bodies, dendrites, and axons in the pedunculus and lobes (Figure 7A). According to their position, these cells undoubtedly correspond to Glu-expressing immature α/βc neurons. Although expression of the GAL4 reporter may, in part, differ from the mGluR pattern, such a precise localization suggests that the new born KCs express the DmGluRA receptor as well.

Twenty-four hours after eclosion, GFP immunostaining is still bright in labeled α/βc cell bodies and axons and individual axonal branches are clearly visible at the end of the α and β lobes (Figure 7B). All around the labeled axons in the lobes, one can notice GFP-positive dots, which may represent sections of smaller axonal branches derived from the main axons (Figure 7B). Ten days later, GFP expression is dramatically reduced in the MB α/βc neurons (Figure 7C). Remarkably, only two bundles out of initially four are still GFP-positive, as can be seen in the calyx and pedunculus presented in Figure 7C. GFP immunoreactivity associated with dendritic fields in the calyx and axons in the lobes is dramatically reduced. In addition, any bright axonal branches and processes have vanished, compared to the staining observed in newly eclosed flies. Such a dramatic change suggests that DmGluRA expression in α/βc neurons is transient and does not persist for long after eclosion. The immunoreactivity still detectable at 10 days might be the remnant of earlier expression and accumulation of the stable GFP protein when the DmGluRA promoter was still active.

### High ubiquitin levels in last-born Kenyon cells of young eclosed flies

**Figure 8:**
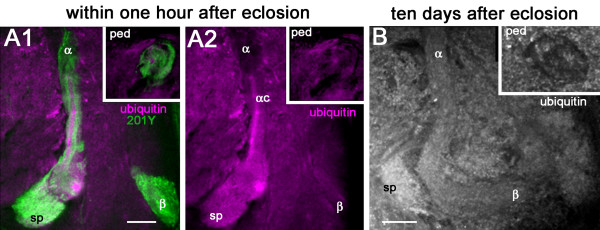
**Ubiquitin immunoreactivity in the MB lobes**. **(A) **Double staining with anti-ubiquitin (magenta) and anti-GFP (green) in brain sections of 201Y-GAL4; UAS-mCD8::GFP. A high level of ubiquitin staining is detected in the α/βc KCs just after eclosion. **(B) **Ten days later, the ubiquitin signal is not bright any more in the core neurons and is distributed all over the MB lobe neuropils, with higher intensity in the spur region (sp). ped, pedunculus. Scale bars: 20 μm.

Ubiquitin is a small protein that binds covalently to specific proteins and either marks them for degradation by the proteasome or modifies their activity [72]. Substrate protein ubiquitination plays important roles in neuronal differentiation [73,74] and synaptic plasticity [75,76]. We found selectively high levels of ubiquitin immunoreactivity in the α/βc neurons of just eclosed flies, as shown in Figure 8A1, A2. The spur region of the γ lobe was also labeled but with lower intensity than the core neurons. In 10-day-old flies, ubiquitin immunoreactivity was found to be homogenous all over the MB lobes, with higher intensity in the spur region (Figure 8B). This result further indicates that the α/βc neurons of newly eclosed flies are in a transient condition of active metabolism related to their maturation.

## Discussion

**Figure 9:**
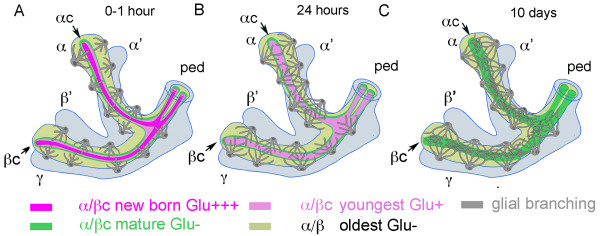
**Dynamics of glutamate expression and glial cell processes in the maturing mushroom body α/β lobe**. The α/β lobe is the last born domain of the MB, formed during pupation. This lobe is entirely covered by glial cell processes that express Glu signaling molecules (dEAAT1, GS). The cell bodies of these glial cells reside outside the MBs. The α/βc consists of: newborn KCs (magenta bright: Glu+++), youngest KCs (pale magenta: Glu+), and mature α/βc KCs (green: Glu-). **(A) **At the moment of eclosion and the first hour thereafter, newborn axons from the α/βc are strongly Glu-positive (Glu+++, magenta bright). These grow down through the surrounding, and older, α/βc neurons: older processes are shown green displaced by the youngest α/βc axons (pale magenta). After eclosion, the only volume of the α/β lobe that does not have glial processes is that in which occur newborn axons of Glu-containing α/βc neurons (bright magenta). **(B) **Twenty-four hours after eclosion, Glu expression in the core neurons has a lower intensity (pale magenta: Glu+) and at the same time delicate extensions of glial cells are seen penetrating into the α/βc. **(C) **Ten days after eclosion: glial cells provide a mesh like network inside the α/β lobes and Glu immunoreactivity is no longer detectable in the core neurons. ped, pedunculus.

One intriguing question in neuroscience is how newborn neurons establish a functional network during their period of growth and maturation. Here we studied in *Drosophila *the late maturation of a subset of the α/β intrinsic MB KCs, the α/βc neurons, during a short period after adult eclosion. In a previous study, Glu-like immunoreactivity was observed in the ingrowth lamina of the cockroach MB, which contains axons of the youngest KCs [77]. Similarly in *Drosophila*, Glu accumulates in the α/βc, which contains newly generated neurons, whereas taurine-expressing neurons were found in the outer α/βc and aspartate-expressing neurons in the rest of the α/β lobes [33]. It has been shown in vertebrates that Glu can have a strong influence on cone motility [78] and induce rapid filopodia protrusion from hippocampal neurites [79] or cultured astrocytes [80]. In the present study, we performed an extensive analysis of the distribution of various glutamatergic markers in the MBs of young adult *Drosophila*. Our results suggest that the α/βc neurons are not simply glutamatergic. Rather, the evidence provided here indicates that these newborn KCs may transiently use Glu as a paracrine agent to favor interactions with glial cell processes and become mature neurons forming functional circuits (Figure 9).

### Glutamate and vesicular Glu transporter

Although the last-born α/βc KCs show a high level of Glu immunoreactivity a few hours prior and after adult eclosion, Glu immunostaining is dramatically reduced in these cells 24 hours after eclosion and is entirely absent a few days later. Disappearance of this signal could result from the release or intracellular metabolism of this amino acid. Similarly, it was observed in cockroach MBs that newborn KCs loose Glu immunoreactivity when they become mature and establish contacts with extrinsic neurons [77,81]. Here we also present the first evidence that Glu transiently accumulates at a high level in developing newborn KCs of *Drosophila *in late larva and during pupal stages. Therefore, transient Glu expression could correlate with KC growth and maturation not only in the α/βc around eclosion time but also in other lobes during earlier stages of MB development.

Three subtypes of vesicular Glu transporters (VGluTs) have been identified in the mammalian nervous system with similar Glu transport functionality [20]. Two of these (VGluT1 and VGluT2) present complementary distribution in central glutamatergic neurons [82,83]. The third isoform, VGluT3, appears to be primarily expressed in neurons that release another transmitter (serotonin, dopamine, acetylcholine or GABA), where it may be required for efficient synaptic transmission [84,85]. In the present study, neither the α/βc neurons nor any other intrinsic MB KCs were found to express the *Drosophila *vesicular transporter DVGluT. This may indicate that the Glu that is accumulated in the inner α/βc neurons is not stored in synaptic vesicles. However, we cannot exclude the possibility that these cells express another vesicular Glu transporter not yet identified in *Drosophila*. We did observe DVGluT immunoreactivity in the MBs, particularly in the γ lobe and spur region and in the α lobes (Figure 1E-J), but the punctuate labeling and localization suggest that this distribution corresponds to glutamatergic synapses belonging to extrinsic neurons.

### Glu transporter and glutamine synthetase

Can the Glu transiently stored in the newborn MB neurons be released into the extracellular space? In the absence of DVGluT or another similar transporter, this could involve a non-vesicular release of Glu. Non-conventional release of Glu from immature neurons has been previously demonstrated in the developing rat hippocampus [21] where Glu release exerts a paracrine action that seems to particularly affect the migration of neighboring maturing neurons [22,23]. To address this question indirectly, we looked for the presence in the MB of other proteins known to be involved in the recycling and degradation of Glu at glutamergic synapses.

An important role of glial cells is to capture Glu released from the synapse with specific transporters and then convert Glu to glutamine with GS. The only *Drosophila *high-affinity Glu transporter, dEAAT1, is expressed in subtypes of glial cells and is associated with Glu-release sites [28,31,86]. GS2 is similarly expressed in glial cells in the *Drosophila *nervous system [70] (T Rival and S Birman, unpublished). We show here for the first time that glial cells expressing dEAAT1 and GS surround the *Drosophila *MB lobe neuropiles, closely enwrapping the α/β lobes, thus isolating them from other lobes, and sending a mesh-like system of extensions inside these lobes. Enwrapping and invading of the MB β lobes by glia was also observed to occur in cockroach MBs, where glial cells are implicated in the removal of degenerating transient KC processes that occur during their establishment of mature connections with extrinsic cell dendrites [81]. The high levels of glial dEAAT1 and GS within the *Drosophila *MB lobes suggest that this neuropil is tightly cordoned off from other parts of the brain and regulates the extracellular Glu level between the axons.

Our data show that GS expression is highly dynamic in the MB during the first day of adult life, suggesting that glial cells play a role in establishing the MB's functional network. During the first hour after eclosion, the meshwork of glial processes expressing Glu signaling-associated molecules (GS and dEAAT1) is not present in the inner α/βc region, but within 24 hours this area becomes covered by glial extensions (Figure 9). These glial elements are possibly guided towards the α/βc area by the gradient of Glu released by the last born KCs. Glia could be involved in reducing Glu concentration in this area and play a role in axonal guidance and final maturation of KCs. Evidence that Glu transporters are required for coordinated brain development has been previously reported for mice: the absence of two glial Glu transporters resulted in excess of extracellular Glu and abnormal formation of the neocortex [87].

### NMDA receptor

Assuming Glu is released by the newborn MB neurons, it has to interact with specific receptors. Therefore, we searched for the expression of Glu receptors in MB neuropiles of young adult *Drosophila*, particularly those receptors that are likely to regulate neuronal growth and maturation through second-messenger pathways. Once activated by simultaneous Glu binding and membrane depolarization, the NMDAR channel allows calcium influx into the postsynaptic cell, where this ion triggers a cascade of biochemical events resulting in synaptic maturation and plasticity [88]. We used available antibodies against the constitutive dNR1 subunit of the *Drosophila *NMDAR [36]. Immediately after eclosion, many processes in the MB neuropil were found to be dNR1-positive, with the exception of the α/βc neurons. The Glu released from either these α/βc neurons, or the surrounding glial cells, or extrinsic MB glutamatergic neurons may activate these NMDAR receptors. Thus, a widespread localization of NMDAR characterizes the MB immediately after eclosion, at the beginning of adult life when the MB is expected to receive the least inputs from sensory interneurons. Subsequently, with increasing sensory data being received and relayed to projection neurons, there is a dramatic and concomitant restructuring of NMDAR signaling: the majority of MB neurons no longer express these receptors. It is only those neurons that receive constant glutamatergic signaling that still address the dNR1 subunit in the vicinity of glutamatergic synapses expressing DVGluT. This occurs in particular within the spur region of the MB and the lateral horn. Such developmentally related regulation of NMDAR expression in the MBs of young adult flies may relate to adaptations of synaptic activity in response to sensory experience [88].

### Metabotropic Glu receptor

mGluRs are neuromodulatory G-protein-coupled receptors that are involved in many aspects of brain physiology, including neuronal development, synaptic plasticity, and neurological diseases [8,89]. Whereas eight distinct mGluRs are present in the mammalian genome, a single functional mGluR is expressed in *Drosophila*, DmGluRA. The fly mGluR is structurally and pharmacologically closer to the mammalian group II mGluRs, which are mainly presynaptic receptors negatively coupled to adenylate cyclase. Our attempts to locate DmGluRA with the commercially available monoclonal antibody 7G11 [90] were not successful because the antibody produced by the hybridoma clone recently lost its binding specificity. To monitor DmGluRA distribution, we used a new GAL4 line that carries an enhancer trap insertion close to the mGluR start site of transcription, keeping in mind that expression of this GAL4 reporter may, in part, differ from the mGluR pattern. Strikingly, the DmGluRA-GAL4 line was found to express GFP selectively in the Glu-accumulating α/βc KCs of newly eclosed adult flies. This is in contrast to commonly used MB GAL4 driver lines (17d-, c739- and 201Y-GAL4) that do not express GFP in these neurons immediately after eclosion. Ten days later, the GFP staining in the DmGluRA-GAL4 line appeared strongly reduced in the α/βc; in contrast, the MB drivers now expressed GFP in these neurons.

Because the GAL4 reporter method reveals whole neurons, we could not determine where the receptor is addressed restrictively in cell bodies, dendrites or axons. A previous study performed with an active lot of 7G11 antibody indicated that DmGluRA is present in nearly all neuropiles of the mature adult fly brain, including the MB calyces, but not in the MB lobes [91]. However, this article did not report on the localization of DmGluRA in newly eclosed *Drosophila*. Further work is required to precisely locate the subcellular localization of DmGluRA in the newborn α/βc neurons, either with a new antibody or a DmGluRA-GFP fusion gene. The source of Glu binding to this mGluR receptor may be the neighboring glial cells or newborn KCs themselves, or both. Through activation of these receptors, Glu is likely to have a transient paracrine action on the α/βc neurons during the first day after eclosion that could be required for dendrite growth or synaptic maturation [92].

Although the α/βc KCs represent a minor part of the α/β lobe neurons, the maturation of these cells appears to be essential for proper MB functioning. Selective expression of the rutabaga (*rut*)-encoded adenylate cyclase in the α/βc neurons with 17d-GAL4 was shown to partially restore olfactory learning and memory in 2- to 5-day-old *rut *mutant flies [49]. In contrast, no rescue of the *rut *defect was observed with c739-GAL4, which expresses in more peripheral α/β neurons at this stage. Therefore, the network involved in olfactory learning and memory apparently requires the α/βc neurons and is already functional in 2- to 5-day-old flies. Furthermore, treatment with mGluR antagonists restored courtship behavior, memory deficits and MB structural defects in *DFmr1 *mutants, a *Drosophila *model of FXS. These positive effects are even stronger when the pharmacological treatment is applied both during larval development and after eclosion [59]. This suggests that these behavioral defects relate to an abnormally high level or prolonged duration of DmGluRA expression in the α/βc neurons of *DFmr1 *mutants [93]. Further study should determine the distribution of Glu and DmGluRA during MB development in *Drosophila *FXS models.

### Ubiquitin

The ubiquitin-proteasome system is one of the major conserved cellular pathways controlling protein turnover in eukaryotic cells. Substrate protein ubiquitination plays important roles in neuronal differentiation [73,74], axonal targeting [94], synapse formation and plasticity [75,76,95]. In addition to strong Glu immunolabeling in the inner α/βc KCs, we also observed a high level of anti-ubiquitin immunoreactivity in these neurons immediately after eclosion. Such a high staining level was no longer detected in 10-day-old flies. In contrast, the spur region of the MB showed a constant high ubiquitin immunoreactivity that did not change with the age of the animal. This could suggest that synaptic plasticity is particularly active in this MB area.

Similarly, labeling of the cockroach MB β lobe with anti-ubiquitin showed, at specific stages in each developmental instar, as well as at an early adult stage, consistent staining of newly generated KC axons. Anti-ubiquitin also labeled the extending transiently Glu-immunoreactive collateral processes from developing KCs in the ingrowth zone, the hemimetabolous homologue of *Drosophila*'s core KCs [81]. These authors showed that ubiquitin expression precedes degeneration of these collaterals and their subsequent removal by scavenging glial cells. Glu receptors can be endocytosed by an ubiquitin-dependent mechanism [75]. The down-regulation of Glu and its receptor protein, possibly mediated by ubiquitin, thus appear to be important steps in the maturation and differentiation of the α/βc KCs.

## Conclusions

The present study suggests that the Glu accumulated in the α/βc KCs of young adult *Drosophila *is used for cell growth and maturation rather than for neurotransmission. The distribution and dynamics of glutamatergic markers indicates that Glu released from newborn KCs can bind to intrinsic mGluRs in the α/βcores and to NMDARs in the rest of the MB neuropil before being captured and metabolized by surrounding glial cells. As an autocrine or paracrine agent, Glu is likely to play a role in pathway finding within the lobe (Figure 9), namely, interactions between maturing KCs and extrinsic neuron dendrites, guidance of glial cell outgrowth and glial process targets into and around the relevant lobes, and maturation of synaptic networks required for a functional MB. Further study of the paracrine function of Glu in wild-type flies and in the *Drosophila *FXS model may shed light on similar actions of this neurotransmitter in the developing human brain in normal and pathological conditions.

## Methods

### *Drosophila *culture and strains

Fly stocks and crosses were maintained at 25°C on standard corn meal-yeast-agar medium supplemented with methyl-4-hydroxy-benzoate as a mold protector. The following strains were used: wild-type Oregon R; UAS-mCD8::GFP to express cell surface membrane-associated GFP [55]; the MB drivers 17d-GAL4 [96], c739-GAL4 and 201Y-GAL4 [97]; dEAAT1-GAL4, which targets Glu transporter-expressing glial cells [31]; and DmGluRA-GAL4, a GAL4 enhancer trap inserted upstream and close to the DmGluRA transcription start site (Y Grau, unpublished). This line was generated by a P-replacement method (see below).

### Targeted transposition

To generate the DmGluRA-GAL4 driver, we mobilized the original *P *element *39C42, w*^+^, localized on the fourth chromosome, 5.94 kb upstream of the translation initiation codon of DmGluRA [24]. Among the white revertant lines analyzed by PCR using genomics primers spanning *39C42*, one deletion event that removed most of the *P *element, including the *miniwhite *gene, but not the *P *element inverted repeats, was recovered; this is called the *P-XVII3:w*^*- *^line. This homozygous viable line was used as a target to isolate replacement events of the residual *P *element by *P(GAL4, w*^+^). *Df(4)O2 *has been described in Bogdanik *et al*. [24]. *Df(4)G(spa*^*-*^) is lethal over *Df(4)O2 *and was obtained from the Bloomington stock center. These and other used mutations are described in FlyBase. The donor strain carrying a *P(GAL4, w*^+^) located on the X chromosome was provided by Dr J-M Dura (CNRS, Montpellier, France).

The *P-XVII3 *enhancer trap targeted transposition screen consisted of the cross of *wP(Gal4, w*^+^); *SbD2-3/+*; *P-XVII3:w*^*-*^*/Df(4)O2 *males to *yw; Df(4)O2/Ci*^*D*^*spa *females. *w*^+^*; Sb+; Ci*^*D *^males were collected and mated to *yw; Df(4)O2/Ci*^*D*^*spa *as single-pair mating. Then, single *w*^+^*; Ci*^*D *^males were crossed to *yw; Df(4)G/Ci*^*D*^*spa *females. Tubes where all *Ci*^*D*^*spa+ *progeny was *w*^+ ^indicated a mobilization of the *P(GAL4, w*^+^) transposon to the fourth chromosome. These lines were established as stable stocks and further analyzed by PCR to determine whether *P(GAL4, w*^+^) had indeed replaced the *P-XVII3:w*^*- *^element. We determined whether the donor *P *element had been replaced in the appropriate location by molecular verification of targeted transpositions events. PCR analysis was performed on genomic DNA with primers specific for the *DmGluRA *gene surrounding the P-insertion site as described in Bogdanik *et al*. [24] as well as primers internal to the *GAL4 *enhancer trap construct (Plac1 and Pry2; described by the Berkeley *Drosophila *Genome Project). All PCR reactions were done as recommended by the manufacturer (Takara, Kyoto). To determine the precise location of the *P(GAL4, w*^+^) insertion in the obtained lines, small regions spanning the junction between the gene and inserted *P *element ends were amplified using PCR and sequenced. We thus found that one line, called *R39-GAL4*, was the result of a targeted transposition event that successfully converted the *P-XVII3 *insertion to a *P-GAL4 *located 5.6 kb upstream of the DmGluRA translation start.

### Western blot analysis

Proteins from *Drosophila *head and dissected brain homogenates were separated on 7.5% SDS-PAGE and transferred onto 0.45 μm nitrocellulose membranes (Bio-Rad Laboratories, Hercules, CA, USA) in 25 mM Tris, 192 mM glycine, 15% methanol at 0.6 A for 2 h at 4°C. Membranes were blocked for 1 hour in phosphate-buffered saline (PBS) buffer containing 10% low-fat powdered milk at room temperature and incubated with mouse monoclonal anti-GS antibodies (Chemicon, Millipore MAB302, Billerica, MA, USA) at 1:2,000 in PBS plus 5% milk overnight in the cold room. Following four 5-minute washes in PBS, membranes were incubated with anti-mouse horse radish peroxidase-conjugated IgG (Jackson Immunoresearch Laboratories, West Grove, PA, USA) at 1:7,000 in PBS plus 5% milk for 2 h. Membranes were washed four times in PBS and developed using chemiluminescence as described by the manufacturer (SuperSignal West Pico Chemiluminescent Substrate, Pierce Thermo Scientific, Rockford, IL, USA). Several exposures were captured on Kodak BioMax MR films.

### Immunohistochemistry

For *in situ *immunostaining, adult male flies of three different ages were collected: either immediately (within 1 hour), 24 hours, or 10 days after eclosion. For each condition, at least three independent experiments were conducted with three to five flies processed each time for brain dissection, agarose sectioning and immunolabeling. The following primary antibodies were used: rabbit Glu antisera and mouse monoclonal anti-Glu (Gemacbio, Saint Jean d'Illac, France; diluted 1:1,000); rabbit DVGluT antisera (gift of Aaron DiAntonio; 1:1,000) [31,33]; mouse monoclonal anti-GS (Chemicon, Millipore MAB302; 1:500) [62]; rabbit polyclonal anti-dNR1 (NMDAR subunit; gift of Ann-Shyn Chiang; 1:500) [36]; rabbit polyclonal anti-ubiquitin (Dako Z0458, Glostrup, Denmark; 1:100) [73]; chicken polyclonal anti-GFP (Abcam ab13970, Cambridge, MA, USA; 1:1,000); rabbit polyclonal anti-DC0 (alias PKA-C1, catalytic subunit of *Drosophila *protein kinase A; gift of Daniel Kalderon; 1:1,000) [98]. The secondary antibodies were used at 1:500 dilution: goat anti-rabbit, Cy5-conjugated (Fab') fragment of IgG (Jackson ImmunoResearch), goat anti-mouse IgG conjugated to Alexa Fluor 555 or goat anti-chicken Alexa Fluor 488 (Invitrogen Molecular Probes, Life Technologies, Carlsbad CA, USA). Preparations incubated in the absence of primary antiserum showed no labeling.

The protocol for Glu immunostaining together with controls of Glu specificity were described in detail previously [32,33,77]. For controls of immunostaining (Figure 1B, inserts), L-glutamic acid (Sigma Aldrich G-6904, Saint-Louis, MO, USA) was conjugated to bovine serum albumin (BSA) via glutaraldehyde Glu-G-BSA following a previously described protocol [99]. After adsorption of the Glu antiserum with Glu-G-BSA (10^-4 ^M with respect to the amino acid), sections of brains showed no labeling. *Drosophila *brains were removed in fixative containing 2.5% paraformaldehyde plus 1.5% glutaraldehyde (both from Electron Microscopy Sciences, Hatfield, PA, USA), 1% sodium metabisulfite (Na_2_S_2_O_5_; SMB; Sigma) in 0.1 M sodium cacodylate buffer (pH 7.0), and postfixed in the same solution overnight at 4°C. The fixed whole brains were incubated for 15 minutes in 0.05 M Tris-HCl, pH 7.5 buffer supplemented with 0.5% SMB (Tris-SMB) and 0.5% sodium borohydride (NaBH4) to saturate double bonds. After a wash in Tris-SMB, brains were embedded in 8% agarose and cut in 60-μm serial sections with a vibratom (Leica). Sections were washed in Tris-SMB supplemented with 0.5% (v/v) Triton X-100 (Tris-SMB-Tx), then pre0incubated with 5% (v/v) normal swine serum (Dako, Glostrup, Denmark) in Tris-SMB-Tx for 1 hour. Brain sections were then simultaneously incubated with rabbit Glu antiserum (1:1,000) and chicken anti-GFP (1:1,000) in Tris-SMB-Tx overnight at room temperature. After a wash in Tris-Tx, sections were incubated overnight at room temperature with conjugated secondary antibodies diluted 1:250. After a final wash in Tris buffer, sections were embedded in 80% glycerol.

For marker proteins, *Drosophila *brains were dissected and fixed overnight in 4% paraformaldehyde in PBS made from tablets (Sigma P4417). The fixed brains were washed in PBS, embedded in 8% agarose and cut in serial 60-μm sections. Sections were washed four times in PBS containing 0.5% (v/v) Triton X-100 (PBS-Tx), then blocking solution was applied consisting of 10% (v/v) swine serum in PBS-Tx. After one night incubation with primary antibodies in PBS-Tx, the sections were thoroughly washed in PBS and then incubated with the secondary antibodies at 1:500. After a final wash in PBS, sections were embedded in 80% glycerol.

### Microscopy

Images were collected on a Zeiss LSM 510 confocal microscope (Carl Zeiss, Oberkochen, Germany). Series of four to ten 0.5-μm optical sections (1,024 × 1,024 at 12-bit color depth) were scanned using 40 × 1.0 or 63 × 1.4 oil iris Plan-Apochromat objectives. The images were stored as TIFF files and edited in Adobe Photoshop CS2. The Glu and dNR1 antibodies gave only superficial staining in sections so the images were composed of two to five optical sections for these antigens. For DVGluT, GS and ubiquitin, images were made from up to ten optical sections from a stack. The contrast and intensity were adjusted accordingly.

## Abbreviations

α/βc: α/β core; BSA: bovine serum albumin; FXS: fragile X syndrome; GFP: green fluorescent protein; Glu: L-glutamate; GS: glutamine synthetase; iGluR: ionotropic receptor; KC: Kenyon cell; MB: mushroom body; mGluR: metabotropic receptor; NMDAR: NMDA receptor; PBS: phosphate-buffered saline; SMB: sodium metabisulfite; VGluT: vesicular Glu transporter.

## Competing interests

The authors declare that they have no competing interests.

## Authors' contributions

IS designed and performed the immunohistochemistry experiments, analyzed the data and wrote the manuscript. YG performed the targeted transposition. NJS supervised initial experiments and wrote the manuscript. SB supervised the experiments and wrote the manuscript. All authors approved the final manuscript.
